# The “Doing” of Compassionate Care in the Context of Childbirth from a Women’s Perspective

**DOI:** 10.1177/10497323241280370

**Published:** 2024-11-19

**Authors:** Carina Vedeler, Anne Britt Vika Nilsen, Soo Downe, Tine S. Eri

**Affiliations:** 1 Research Group Midwifery Science, Faculty of Health Sciences, 60499 Oslo Metropolitan University, Oslo, Norway; 2 Department of Health and Caring sciences, Faculty of Health and Social Sciences, 366044 Western Norway University of Applied Sciences, Bergen, Norway; 3 ReaCH Group, School of Nursing and Midwifery, 6723 University of Central Lancashire, Preston, UK

**Keywords:** compassionate care, midwifery, childbirth, quality of care, maternity care, intrapartum care, qualitative methods, qualitative interviews, women’s experiences

## Abstract

Women who are giving birth need to be met with compassion and understanding from healthcare professionals. However, there are growing concerns about the perceived lack of compassion in the delivery of healthcare services in general and maternity care in particular. We conducted 15 qualitative interviews with women who had given birth in Norway within the previous year, asking them to describe their experiences of compassionate care. We aimed to explore what healthcare professionals “do” that is experienced as compassionate. The analysis was informed by Paul Gilbert’s theory of compassion and a concept analysis of compassionate midwifery undertaken by Ménage and colleagues. The compassionate caring actions of healthcare professionals that were identified in the women’s narratives generated five themes: attuning actions, validating actions, contextualizing actions, empowering actions, and small acts of kindness. The findings build on the prior theoretical concepts used for the study and provide a nuanced account of how women perceive compassionate care from healthcare professionals. They could contribute to understanding more of the meaning and nature of compassionate care during childbirth. The analysis indicates the importance of ensuring that compassionate care is at the very core of maternity care services.

## Introduction

There is extensive evidence that women who are giving birth need to be met with compassion and understanding from healthcare professionals (Ménage et al., 2017; Renfrew et al., 2014; Vedeler et al., 2022). Furthermore, the positive implications of a compassionate healthcare system have been widely recognized in both research and guidelines (International Federation of Gynecology And Obstetrics et al., 2015; Malenfant et al., 2022; Sinclair et al., 2016). Despite this, over the past decade, international research and reports have increasingly raised concern about the perceived lack of compassion in the delivery of healthcare services in general (Malenfant et al., 2022; Tehranineshat et al., 2019) and maternity care in particular (Bohren et al., 2015; Kirkup, 2022; Vedeler et al., 2023).

Giving birth is a pivotal life-changing event that can be considered one of the most significant transitional phases in the lives of human beings. The transition to motherhood involves emotional, social, and physical implications crucial to future health and well-being of women and families (Renfrew et al., 2014; WHO, 2018). Hence, the experiences of care and support during this period hold immense importance for both the mother and the baby and her family (Lundgren et al., 2009; Meyer et al., 2022). Additionally, meeting women’s needs during childbirth may contribute to the health-promoting potential of childbirth, providing families the best possible start (Downe et al., 2018; Finlayson et al., 2020). In comparison, a lack of compassionate high-quality care during labor and birth can lead to poorer physical and psychological health and well-being, such as difficulties in the transition to motherhood (Beck & Watson, 2008; Bell & Andersson, 2016), increased anxiety and depression (Alcorn et al., 2010; Kranenburg et al., 2023), and negative and traumatic birth experiences (Koster et al., 2020; Reed et al., 2017). These experiences can also have negative consequences for further reproduction (Shorey et al., 2018).

### Compassionate Healthcare

According to the Oxford English Dictionary (2023), “compassion” stems from the Latin “compati,” meaning “to suffer with.” Although there are a range of various definitions of compassion, there appears to be a broad consensus that compassion entails both a *sensitivity* toward others’ suffering and distress and *motivation* to act on this (Gilbert, 2020; Nussbaum, 2001; Shea et al., 2014).

Within the healthcare community, compassionate healthcare is commonly defined as “an emotional state and its associated behaviours as recognition, understanding, emotional resonance, and empathic concern for another’s concerns, distress, pain and suffering, coupled with their acknowledgement, and motivation and relational action to ameliorate these conditions” (Lown, 2016, pp. 333–334). This implies that compassionate healthcare entails empathy, sympathy, and genuine concern for the person’s suffering or distress, as well as a willingness to act to meet the person’s needs. However, cultural norms and values can affect what is perceived as compassionate healthcare (Singh et al., 2018). For example, in some cultures, maintaining eye contact or providing physical comfort might be seen as compassionate while in other cultures, such actions might be deemed as intrusive or inappropriate (Babaei et al., 2016; Singh et al., 2018).

Although compassionate care has been shown to have multiple benefits, including improved health outcomes and satisfaction with care provision and well-being (Malenfant et al., 2022), there are few studies exploring compassionate care in the context of childbirth and maternity care services. In a recent study of more than 8000 women’s experiences of care during childbirth in Norway, one of the main findings was the importance of compassionate and respectful care from healthcare providers (Vedeler et al., 2022). Other studies, among them one undertaken in Norway (Vedeler et al., 2023), have shown that a lack of compassionate care is at the core of women’s negative and even traumatic childbirth experiences (Koster et al., 2020; Meyer et al., 2022).

In line with the compassionate healthcare definition, compassionate midwifery is defined as “the interrelations of authentic presence, noticing suffering, empathy, connectedness/relationship, emotion work, motivation to help/support, empowering women and alleviating suffering through negotiation, knowledge and skills …” (Ménage et al., 2017, p. 558). Moreover, the authors argue that compassion is a new and evolving concept within the field of midwifery, warranting further exploration because of its capacity to enrich both the theoretical foundation of midwifery and its practices (Ménage et al., 2017). They argue that the notion that compassionate midwifery is something all midwives comprehend and are familiar with could be presumptive rather than inevitable, and that there is limited understanding of whether women and midwives have a shared conception of what constitutes compassionate care (Ménage et al., 2017).

To our knowledge, only one study (Menage et al., 2020), undertaken by the same team that proposed the definition of compassionate midwifery, has explicitly explored women’s lived experiences of compassionate care in the context of childbirth. The study, focused on midwifery care, demonstrated that women perceive compassionate midwifery to encompass three key themes: providing presence, fostering relationships, and empowerment. While Menage et al.’s (2020) study provided important insights into compassionate midwifery care, a broader exploration focusing on healthcare professionals’ compassionate *actions* is needed.

### Aim

Given the background, the aim of the present study was to contribute to the knowledge base about compassionate care in the context of childbirth. We raised the question of *How do healthcare professionals “do” compassion?* This included an exploration of what and how women recognize, describe, and experience healthcare professionals’ compassionate caring actions and behaviors, conceptualized as the “doing” of compassionate care. By this, we aimed to explore and describe in depth compassionate maternity care from women’s perspectives.

### Theory, Physiology, and Psychology of Compassion Framing the Study

The current study is informed by Paul Gilbert’s theory of compassion (Gilbert, 2009, 2014, 2017, 2020) and Ménage et al.’s (2017) concept analysis of compassionate midwifery. Gilbert’s conceptualization of compassion provides a comprehensive understanding of compassion from both an evolutionary and psychological perspective and shows how compassion is an essential part of human nature (2009, 2014, 2017, 2020).

Gilbert (2014) defines compassion as “a sensitivity to one’s own and others’ suffering or distress with a deep commitment to try to alleviate and prevent it” (p. 19). He notes that compassion is often confused with other relatable emotions, such as pity, sympathy, and empathy, and it is commonly reduced to characteristics such as warmth, kindness, and gentleness. According to Cole-King and Gilbert (2014), these are necessary but not sufficient characteristics, since “compassion is much more than that” (p. 69).

In evolutionary psychology, compassion is considered a basic social emotion and an evolutionary premise for maintaining social boundaries, staying close, and dealing with negative affect (Cole-King & Gilbert, 2014; Gilbert, 2017). However, according to Gilbert (2017, 2020), human social intelligence enables caring and compassion to extend beyond kin and reciprocal relationships because it also includes knowing awareness and capacity for metacognition—the ability to think about thinking.

Furthermore, compassion entails hormonal processes related to caring (Gilbert, 2017, 2020; Uvnäs-Moberg et al., 2019). When both *giving care* and *receiving care,* oxytocin and endorphins play central roles. Receiving certain kinds of care raises oxytocin levels, has a calming effect on the amygdala, and provides a sensation of safety and well-being (Gilbert, 2017). Soothing touch produces endorphins and oxytocin, promotes the parasympathetic nervous system’s relaxing characteristics, and decreases cortisol (Gilbert, 2020; Uvnäs-Moberg et al., 2019).

The psychology of compassion is a complex process that includes a combination of both attributes and actions (Gilbert, 2009, 2017, 2020). According to Gilbert (2009, 2017), the essential attributes for compassion involve sensitivity to one’s own or others’ suffering, including the ability to engage with and comprehend the suffering. The associated skills for compassion encompass appropriate actions and the motivation to alleviate or prevent distress, along with the competence to do something about the suffering (Gilbert, 2009, 2017).

In the previously cited concept analysis of compassionate midwifery, a synthesized model shows how compassion is not just a moral position or trait but must be understood as a process that links emotions, motivations, and actions (Ménage et al., 2017). The four-step process model proposed consists of *a recognition* of another’s suffering, followed by *an emotional response* or *connection* with the sufferer, which leads to *the motivation* to try to alleviate the others’ suffering and then *actions or behaviors* aimed at alleviating it (Ménage et al., 2017).

## Methods

### Design

This study has a qualitative design with an experiential approach. Experiential qualitative research explores the participants’ experiences, views, perspectives, and practices (Braun & Clarke, 2021, 2022). Although we wanted to keep an open mind to explore women’s experiences of what they perceived as healthcare professionals’ compassionate caring actions and behaviors, the theory of compassion served as a guiding conceptualization throughout the analysis to better understand the complexities and nuances of compassionate care. We used qualitative in-depth interviews to explore women’s experiences of the “doing” of compassionate care (Brinkmann & Kvale, 2018).

### Context

The context of the current study was the maternity care system in Norway. In Norway, midwives attend all births and have the main responsibility for women during labor and birth, if everything proceeds without complications. If there are any risk factors to the pregnancy or during labor and birth, there is a team of midwives, doctors, and nurse assistants who care for the woman. On the postnatal ward, there are mainly midwives, nurses, and nurse assistants, in addition to pediatricians, who examine the new-born. Obstetricians are on call, if needed. The maternity care system is part of the public health system, which is tax funded and free of charge. There are obstetric units, alongside midwifery units, and free-standing midwifery-led units, while homebirths are assisted by independent midwives. Despite an emphasis on high-quality care within the maternity system in Norway, some women report experiences of uncompassionate and disrespectful care during childbirth (Lazzerini et al., 2022; Reppen et al., 2023; Vedeler et al., 2023).

### Recruitment and Data Collection

A purposive sample of 15 women who had given birth in Norway was included. The only inclusion criteria were that they had given birth within the last year and that they were more than 18 years old. While our study was inclusive of diverse backgrounds and birthing experiences, some questions relating to sociodemographic backgrounds were not asked because of the cultural norms in Norway.

We used the snowball approach to recruit participants, and those interested in participating received an information letter describing the study. The first participants were recruited through a maternity care user organization. Each respondent was asked to recommend to the researcher another woman who might be able to articulate their views about their experiences of compassionate care during childbirth. The final numbers of participants were guided by the concept of “information power,” which suggests that the more information the participants hold relevant to the study, the fewer participants are required (Malterud et al., 2016). Based on this, the final number was determined when the collection of data was deemed to have enough “information power” to address the aim of the study.

The participants could decide whether they wanted to do the interview virtually or if they wanted to meet in person. This was with the intention of making it feasible for the women to participate and still take care of their baby (Hanna & Mwale, 2017). All the women preferred to have the interviews virtually. For some, this was due to the COVID-19 pandemic, others lived a long way from the research site, and others preferred on-line interviews for personal convenience. The interviews were undertaken by the first author. They took place between April 2022 and February 2023 and lasted between 60 and 90 min. All the interviews were audio-recorded using the Dictaphone app, with the permission of the women interviewed, and the recordings were professionally transcribed verbatim. Additionally, the immediate impressions and interpretations of the interaction were noted by the researcher and discussed within the research group. One of the interviews was conducted in English, while the rest were in Norwegian.

The interviews had an open exploratory approach. The first author started by informing the participant about the focus of the study. First, they were informed that women’s need for compassionate and respectful care was one of the major findings of a prior study performed by the research team about important aspects of care during childbirth (Vedeler et al., 2022). Then they were told that, in the present study, the research team aimed to explore this further by asking for detailed descriptions of healthcare professionals’ actions and behaviors that were felt to be compassionate, and what the emotional response to these actions and behaviors might be. The participants were asked to provide accounts of situations where they recognized either compassionate care or a lack of compassion. Through this approach, the team aimed to facilitate reflections on specific, impactful situations of compassionate care, thus enabling the participants to describe these situations in detail. They were also encouraged to freely describe and reflect on their labor and birth experiences. This open approach was designed to gather contextual information about the women’s experiences. Probes included *please tell me more about that, how did it make you feel, could you try to describe what happened, how did the healthcare professionals behave and act, what did they say*, and so forth.

### Reflexive Thematic Analysis

We applied a reflexive thematic analysis, as described by Braun and Clarke (2021, 2022). Throughout the analysis, the theory of compassion guided us in exploring how women identified and experienced compassionate caring. We sought to distinguish and recognize examples of healthcare professionals’ sensitivity to “suffering,” and their motivation to alleviate and prevent the “suffering” in the women’s accounts. To grasp the meaning of compassionate care in the women’s narratives, we searched for how compassionate care was manifested and articulated.

The analysis followed six phases: the first phase, becoming familiar with the data, involved searching for descriptions that could be interpreted as a recognition of healthcare professionals’ compassionate caring actions. The first and last authors read through all the interview transcripts to obtain a good overview of the data; similarities, differences, and interpretations across the participants’ accounts were noted by both independently. The second phase involved generating initial codes to capture broad descriptions of compassionate care. In the third phase, the first author searched for themes representing what women recognized as specific compassionate caring actions. In the fourth phase, the first and last authors reviewed the generated themes; we consciously considered whether the themes answered the research question and provided meaningful interpretations and coherence of the data. The fifth phase included the process of defining and naming the themes through discussion among all the authors, while the sixth phase consisted of writing the report.

The analytical process was iterative, which involved moving back and forth between the data, codes, generated themes, and the theory of compassion. During this process, we were conscious of not using the theory as a predefined framework but rather as a guidance to comprehend more of the significance of compassion in the women’s narratives and to enhance our understanding of the nuances in the themes (Malterud, 2016).

### Rigor and Reflexivity

Rigor and reflexivity were maintained through a systematic and iterative approach to data collection and analysis, in addition to a conscious use of the theory of compassion and by adopting a reflexive approach. By performing a reflexive thematic analysis (Braun & Clarke, 2021, 2022), we ensured various levels of data exploration, including familiarization, coding, theme generation, theme revisions, and report writing. This process allowed us to deeply engage with the data and contributed to a thorough exploration and interpretation of the women’s experiences of compassionate care. Rigor was further ensured through the iterative nature of the analytical process, which involved constant movement between data, codes, themes, and the theory of compassion, to ensure that the interpretations were grounded in the data (Braun & Clarke, 2021).

In qualitative research methods, the researchers inevitably influence the research process (Braun & Clarke, 2021; Malterud, 2001). A reflexive analytical approach implies a reflexive engagement by the researcher throughout the research process (Braun & Clarke, 2021). Our research team consisted of a PhD student (CV) and three PhD-qualified senior researchers (TSE, ABVN, and SD), all of them experienced midwives, researchers, and mothers. Based on our experiences and former research, we believe that childbirth can be a transformational experience for women, parents, and babies that can affect them, positively or negatively, for the rest of their lives. We also believe that the way maternity care staff (midwives, doctors, and others) interact with maternity service users and their birth companions is critical to both their short- and longer-term well-being. Nonetheless, although the research team shared many of the same positions regarding childbirth, we also have different backgrounds. One of the researchers is from the United Kingdom, whereas the rest are from Norway. Furthermore, the team hold different research and clinical experiences form various contexts.

To maintain reflexivity, the first author wrote memos and we continuously critically discussed codes and potential themes. Through this reflexive process, we aimed to derive meaningful interpretations of the data (Braun & Clarke, 2021), guided by the theory of compassion, contributing to new insights about the concrete “doing” of compassionate care.

### Ethical Considerations

The study was carried out in accordance with the Helsinki Declaration of Medical Research. The Norwegian Data Protection Services for research (Sikt) assessed the research protocol to ensure our research data processes complied with ethical and legal requirements and approved the study (no. 447156). The Regional Committee for Medical Research Ethics (REC south-east) assessed the research protocol and concluded that it was exempt of approval (no. 440977) because it did not involve risks for the participants.

All participants received both oral and written information about the study and about their right to withdraw at any time with no further consequences. Oral consent was obtained and recorded by all the participants before the initiation of the interviews. All participants were told that interview data would be treated confidentially. Data were anonymized using identification codes. The sound-recorded interviews and personal information were stored in a service designed to protect sensitive data, The Services for Sensitive Data (TSD), managed and provided by the University of Oslo.

## Results

In total, 15 women between 29 and 41 years old took part in the study. Five of the women were primiparous, and 10 were multiparous (three of them had given birth to their third child). Two of the women had a planned homebirth, one had an unplanned homebirth, two had given birth at an alongside midwifery unit, while the rest had given birth at an obstetric unit. The sample encompassed various birth experiences, including different modes of birth, medical interventions, and complications. The time since birth was 1.5–10 months (mostly between 2 and 4 months). All the women had partners. Two of the women were not born in Norway, and one had a partner who was not born in Norway.

Five themes were generated through the reflexive thematic analysis: *Attuning actions: situational attentiveness; Validating actions: you matter; Contextualizing actions: situational framing; Empowering actions: feeling encouraged; and Small acts of kindness: small yet important*. The five themes were interpreted as a sense among respondents that “doing compassion” involved a balancing act for health professionals between being sensitive, loving, tender, soothing, and comforting, at the same time as having the courage and ability to act, when necessary, without hesitating, yet maintaining respect and kindness. Although we did not explore differences in experiences of compassionate care across providers, most of the interactions referred to in the results included midwives due to the structure of the healthcare system in Norway. However, the results also include interactions with obstetricians and nurse assistants.

### Attuning Actions: Situational Attentiveness

The concept of “Attuning actions” captured women’s accounts of encountering healthcare professionals as sensitive and tuned into the woman’s situation. It implied experiencing healthcare professionals as being aware, attentive, and focused on women’s needs, which made them feel genuinely “seen.”

Tuning into the woman could involve a soothing calming voice, a tender look, a friendly gentle touch, or dimming the light to attune into the woman’s state of mind. Through these acts, healthcare professionals showed that they were attentive and adaptable to a woman’s situation and that they genuinely cared about their emotional state and needs in that specific situation.

It was also so nice that it was quiet, and it was so calm; there was almost no talking. So, there was a lot of respect in the delivery room. I kind of just got to be in my own birth bubble.

This sensitivity toward the specific woman’s individual needs was vital. Some women described needing a tranquil and peaceful ambience in the room, while others described how a cheerful, light atmosphere and humor were experienced as pleasant and uplifting.

When healthcare professionals seemed approachable, used clear and understandable language, and involved the woman in the conversation, it was easier to create meaningful connections and was perceived as inclusive. In contrast, dense medical language and Latin words could make the women feel that the healthcare professionals were detached and distant and that they did not pay attention to whom they were speaking.

She [the obstetrician] was absolutely wonderful. There was something about the personal chemistry with that doctor. Because she was so jovial and kind of easy to talk to, I felt that we understood each other. And she somehow didn’t use the medical language.

Experiencing the midwife or doctor being attuned to their state of mind was also revealed through the balancing act of feeling reassured but at the same time not feeling disturbed. The notion of the midwife’s watchful attendance and attentiveness, that she was present and available if needed, implied that the woman just knew that she was there.

When I opened my eyes, they were always there, in a way I could always ask, and I always could. Even though I didn’t do it, I knew I could if I needed to.

This perception of sensitivity and respectfulness toward the woman was described by the women as “not needing to say anything” and “the midwife just knew what I needed.” Others described how they felt reassured just through a silent confirmation, a small nod, a smile, or through eye contact.

Without it being said in words, I still received confirmation that ok, but it’s okay, it’s right, what I’m doing is okay. And it’s very reassuring in a way that you don’t get comments on what you do or sounds you make that might feel strange, so I could kind of look at them and they gave me a very clear impression that this is normal. In that way, I didn’t have to ask questions with words. I could just look at their faces and know it was okay.

The women described in various ways how they appreciated it when healthcare professionals showed how they had time for them and wanted to accommodate their needs in the situation. This could be by entering the room or sitting down by their bed instead of standing in the doorway, thereby showing that they were “present” rather than just seeking information or checking up. It also involved them spending time with the woman, being in the room when she breastfed, or helping her attend to the new-born.

No matter what you ask, it’s like: we’ll sort it out. I don’t think I ever felt like they were in a hurry. I did hear the alarms and the phone, but I didn’t notice them. I think it’s impressive that they manage to keep calm in the conversation, even if they were short on time. They entered the room and were so present.

Another woman described a situation when a nurse assistant was there together with her when changing the baby’s diaper and how the nurse assistant held the baby’s hand and gently stroked the belly. Through this action, the nurse assistant showed that she was tuned into the woman and her baby by showing an interest in *them* and by sharing the moment with *them*:

… and when I changed the nappy and she came in, she holds the baby in … or her finger in her hand and strokes the belly a little and smiles and … again then, maybe reflects a little my pride and a bit like that recognition that, yes, this is a beautiful child.

### Validating Actions: You Matter

Validating actions included interactions that made the woman feel seen, heard, and recognized. This could include simple gestures and efforts such as using the woman’s name, giving compliments, sharing their happiness, and a genuine interest in understanding and accommodating the woman’s experiences and expressions. When healthcare professionals used the woman’s name, it was perceived as an explicit act in which she felt “seen” and recognized as a particular person with value. Feeling validated also included healthcare professionals explicitly asking about expectations and wishes and saying or showing through their behaviors that they had read their medical records or birth plan.

I remember the midwife talking about the birth plan and mentioned such simple things that I had written. It made me feel that they wanted to sort of take care of me and hear what I had to say. I got the feeling that I wasn’t just one in a row, but it was important for them to know exactly what I wanted to say and how I wanted my experience to be.

Not having to promote their own expectations and wishes but noticing that the midwife or doctor remembered what was said or what they had agreed was highly valued.

Having a postpartum talk with the midwife or obstetrician was important to experience closure in relation to the childbirth episode. Some interviewees emphasized the benefit of having staff who asked open questions about their experiences and who showed an interest and attention into how *they* had perceived or interpreted different situations. By listening thoroughly and confirming whatever experiences and emotional reactions she or her partner expressed, healthcare professionals showed an ability to tolerate and contain their reactions, emotions, or situational understanding instead of avoiding or diverting the woman’s reactions. It seemed vital that the healthcare professionals were able to really listen without having their own agenda or a need to inform or correct the woman’s narratives. Through these kinds of encounters, the healthcare professionals showed how the woman’s and her partner’s experiences mattered and were important for them to understand.

I think that she at the hospital who came in at the maternity ward was very like that … the best thing about her was that she just listened to everything I wanted to say and kind of agreed … that she didn’t seem like she was in a hurry, she seemed not like this was just something she had to do. She sort of sat down properly. Yes, she had plenty of time.

Yet another way of validation was through involving the partner or others present, for example, a doula, as shown in the following quote:

It was very important to me that there was such mutual respect between the midwife and doula. The way they communicated with each other, yes, the midwife could also ask the doula questions—it wasn’t like that I am the midwife and I know everything, and you are below me in a way. For example, what do you think, how far has she come now do you think?

By creating an inclusive and mutually respectful environment, the healthcare professionals showed that they were adaptable to what mattered to that specific woman and were aware of their responsibility and ability to contribute to a respectful environment.

### Contextualizing Actions: Situational Framing

Contextualizing actions and situational framing refer to healthcare professionals’ concrete actions, which contributed to making a situation more understandable and manageable. These actions could involve speaking directly to the woman, looking her in the eyes, using her name, calming the woman, and taking immediate action without any form of hesitance or doubt, while making sure this was done respectfully, and while ensuring she understood what was happening and why, and that she had some control over the process.

The women described feeling an immediate difference when specific healthcare professionals entered the room for the first time. They referred to the notion of them taking control in a particular situation and acting clearly without hesitance or doubt. This could be achieved by using a clear and firm voice and touch, which showed there was no trace of doubt or avoidance, here showing that they knew what they were doing and were in control. One woman said, “She was hands on—and told me everything she did.”

While such certainty could be experienced as controlling and condescending, what made it an act of “doing compassion” in these accounts was a fine-tuned balance between generating confidence through demonstrating control and agency and being sensitive to and respectful of the woman’s needs, values, and choices. Experiencing this sensitivity together with trust, that the healthcare professional would act if this was needed, led to a feeling of safety.

On the other hand, feeling that healthcare professionals seemed uncertain, hesitant, or doubtful in their actions could contribute to a sense of feeling unsafe, which detracted from the experience of being met with compassion. In this case, rather than empathizing, and relieving suffering, the experience was of a lack of empathic engagement and an increase in suffering.

She was a little stressed, I think, which also stressed me a little, because I think she was afraid that I would get preeclampsia. I was affected by the fact that she kept saying I had high blood pressure.

The women emphasized the need for feeling safe when something did occur that they did not understand or when things became chaotic. When healthcare professionals explained what happened or defined and normalized the situation, this was perceived as calming and created coherence for the women. Furthermore, this contributed to creating context and could lead to making the situation understandable and manageable for the woman. This led to a sense that their suffering was understood and ameliorated.

The midwife and doula very quickly said that this is completely normal, just try to breathe and we will help you, and they supported me and got me dry so that I didn’t get so cold and helped me and lie down on the sofa and wrapped me in blankets and such. It was very good to have that normalisation. It is completely normal. Many people experience this; nothing is dangerous.

The women also described how they experienced situational framing through not feeling condemned, ashamed, or rejected when feeling vulnerable and dependent on those around them. This implied not feeling alone when experiencing being vulnerable and helpless; it also involved healthcare professionals being able to contain and tolerate the situation.

One woman provided an account of being naked and vomiting in the bathroom while the midwife was just there, being supportive and staying with her, without making any negative comments. This led to the notion of not feeling judged but instead feeling emotionally embraced by the midwife.

I was completely naked in that delivery room then, and I’m sweating and crying and throwing up and having to pee … so … yes, it was … there’s a lot of body and a lot of stuff. And I never, for a second, felt like they were judging me or looking down on me or thought it was disgusting or anything. That’s very good. Now, it would have been terrible if it had been like that, but it may have been that some people felt that way. I thought about that afterwards, that … you’re never that vulnerable. And especially being naked, that’s something … Throwing up too, it’s very … And very discrete, but very warm, at the same time in the whole thing.

In contrast, a throwaway comment about a sensitive issue could lead the woman to feel disrupted and embarrassed as the quote below shows:

And then he was in the opening, and then, there was faeces in the bathtub, and she said that … she said something about it, that there was a lot of faeces, so she wanted to take it away then. OK, there were faeces in the bathtub, but how dangerous is that really … I needed her to say this is going well, you’re doing exactly what you’re supposed to do.

### Empowering Actions: Feeling Encouraged

Empowering actions refers to healthcare professionals being encouraging, supportive, and giving compliments and praises. This was emphasized as an important part of their genuine caring engagement in “doing” compassion.

They cheered me. I liked that with all three midwives, that they cheered me a lot. They also gave me praise. I think all the midwives I’ve had in the last phases have been like that … it seems like you’re running a marathon, and then, they say you’re the best and you’re so good at this. It feels so good to get such feedback.

Such encouragement led the women to feel strong, supported, and proud and was described by the women as incredibly empowering. They explained how healthcare professionals made them feel like *the strongest woman ever* when receiving compliments and praises. Getting compliments and hearing beautiful things about the baby from a midwife or doctor made a significant impact and was considered as more important than getting a compliment from anyone else because “they knew what they were talking about.” Some respondents were aware that healthcare professionals presumably gave compliments to everyone, but the way it was done meant that it was still experienced as being authentically individualized to them, making them feel special and unique.

One woman who had an unplanned homebirth described coming into the hospital after the baby was born. She felt moved by everyone who greeted her with congratulations: “it was as if everyone knew I had given birth at home,” and she described how this made her feel full of pride.

### Small Acts of Kindness: Small Yet Important

The women emphasized the small, sometimes almost invisible, yet important, reassuring, well-intentioned, and kind acts that led to feeling cared for and created a welcoming and friendly atmosphere, as one woman described: “They were so loving and welcoming. I just felt that she was so kind and helpful.”

The small acts of kindness included healthcare professionals’ friendly appearance, a welcoming smile, responsive looks, comforting words, compliments, supportive language, helpfulness, and understanding. This could also involve practical aspects of “doing” compassion, such as bringing food and drinks, and generally being helpful and kind.

There were also things like her making dinner for us after the birth and cleaning and changing our bed and running the dishwasher and doing practical things like that.

Through these small acts, healthcare professionals showed that they intended to make the labor, birth, and postnatal experience as comfortable as possible for the woman and her partner through anticipating and minimizing any possible suffering, and that the staff were concerned for them as unique and respected individuals. The experience that staff members were willing to put in extra effort to care for them created a notion of being genuinely cared for.

The midwife who attended the birth came to the postnatal ward. She did it on her own initiative; it wasn’t a routine, but it was just something she wanted to do, perhaps a need for her own part as well. I felt like she cared; it wasn’t just a shift at work; it wasn’t just yesterday; we’re done with that. It was people who were important.

Small acts of kindness also involved tactile care, a comforting touch, warm hands, massage, or giving a cold or warm compress.

The nurse assistant, I have to say that she was incredibly nice. She was so encouraging and so very warm and held my hand and touched me like this. She touched my forehead, wiped away the sweat.

She massaged my lower back, dipped a towel in water and placed it on my lower back. (I got that heat, and things like that.) It was absolutely fantastic.

The tactile care was highly appreciated and emphasized as something that appeared as a memory that stood out, a detail that was remembered. It was described as something that contributed to a relaxed and comfortable atmosphere—a bodily experience of soothing care.

## Discussion

In this current study, we have explored women’s experiences of compassionate care during childbirth in a Norwegian context. Drawing on conceptual theories of compassion as our foundation, we have explored what compassionate care entails in the context of childbirth, how the “doing” of compassionate care is perceived and recognized by women, and how healthcare professionals can act compassionately when encountering women during childbirth.

Compassionate caring actions identified in the women’s narratives generated five themes: *Attuning actions, Validating actions, Contextualizing actions, Empowering actions, and Small acts of kindness*. Our findings show that the women experienced compassionate care when healthcare professionals were both sensitive and adaptive to the woman’s signals, needs, and vulnerability while showing the ability to tolerate emotions and act upon whatever was needed. The findings broadly mirror the concept analysis of compassionate midwifery (Ménage et al., 2020), which shows that compassionate midwifery involves the balancing act between being both sensitive to the woman’s needs and vulnerability and a motivation to try to remedy her needs. In line with Gilbert’s theory of compassion (2009, 2017, 2020), the compassionate actions described in the five themes incorporate both a sensitivity and warmth toward the woman’s needs and a motivation to alleviate them through concrete “doing” of compassionate actions.

Although other studies have highlighted the need for individually adapted care (Leinweber et al., 2022), our findings provide more details about the responsive nature of compassionate care. The findings may contribute to an enhanced understanding of how personalized care may be performed and could guide healthcare professionals in how to adjust their approach in response to each woman’s specific evolving situation and emotional state.

An example of how healthcare professionals acted compassionately was when they lowered their voice, which demonstrated and manifested their sensitivity and attunement to the specific situation. Attuning actions also involved being attentive and present, for example, when entering the room, where they sat in the room, and by showing that they were there, if needed. This is in accordance with the concept of watchful attendance, describing the distinctive presence of a midwife in the room (de Jonge et al., 2021). Through the validating actions, women described how they felt seen and respected and how they also got a sense that their experiences mattered and that their stories and emotional expressions were tolerated. Studies have shown how important it is for women to feel that they are respected and listened to during childbirth (Koster et al., 2020; Reed et al., 2017; Shakibazadeh et al., 2018) and, furthermore, how asking for women’s views and permissions are fundamental for the experience of self-determination (Ebert et al., 2014; Heys et al., 2021). Yet another part of compassionate actions was the contextualizing actions, which were important for feeling in control and being able to trust the healthcare professional’s competence. The importance of trust in being able to feel safe has been found in several other studies (Vedeler et al., 2022; Werner-Bierwisch et al., 2018).

While the attuning, validating, and contextualizing actions may be interpreted as fundamental parts of compassionate care, two more actions were highly appreciated and highlighted as significant by the women: empowering actions and small acts of kindness. The accounts of specific empowering actions indicated a genuine commitment to the woman as an individual by the healthcare professional, supporting here to feel unique and strong. Other studies have shown that women perceive caring encounters during labor and birth as empowering (Leinweber et al., 2022). To feel empowered during childbirth may contribute to women feeling a sense of control, enhancing their confidence and capability in their new role as mothers (Downe et al., 2018; Finlayson et al., 2020). This may further highlight the health-promoting potential of compassionate care, suggesting that compassionate care can have a positive, long-term impact on a woman’s health and well-being in the transition to motherhood.

Furthermore, small acts of kindness served as forms of “doing” compassion by creating a calming, friendly, and pleasant atmosphere or by a caring touch. Studies have shown how a caring touch has a therapeutic effect because it can bring comfort and calm to patients in general, beyond maternity care (Karlsson et al., 2022), and specifically that it may decrease labor pain and anxiety (Pinar & Demirel, 2021). However, cultural differences can influence how actions like eye contact or touch are interpreted (Singh et al., 2018). For instance, in some cultures, touch may be deemed acceptable only when performed by healthcare professionals of the same sex as the patients (Babaei et al., 2016). In this case, “doing” compassionate care would imply tailoring small acts of kindness to the cultural as well as personal values, beliefs, and norms of each woman.

### Implications

The women’s descriptions of the general nature of compassionate care in this study correspond with Gilbert’s theory (2009, 2017, 2020) and with the findings of Ménage et al. (2017, 2020). They also fit with how midwives are reported to perceive compassionate midwifery (Krausé et al., 2020). However, our study goes beyond this general conceptual framework because it provides details on what healthcare professionals actually do that is experienced as compassion. Detailed knowledge and understanding of how healthcare professionals can act compassionately is fundamental to meet the needs of women during childbirth, as reports on women’s experiences with maternity care services continue to reveal failures of compassion, in Norway and elsewhere (Kirkup, 2022; Ockenden, 2022; Vedeler et al., 2023), and studies show that uncompassionate care is at the core of women’s negative experiences with maternity care services (Koster et al., 2020; Meyer et al., 2022; Vedeler et al., 2023).

According to Ménage et al. (2017), it is problematic that compassionate midwifery is perceived as either practical or emotional because this creates a false separation. This understanding also comes to light through the idea that one can distinguish between medical treatment and emotional care. Based on our findings, we argue that it is the combination of these two aspects that women value, which is also in line with what is perceived as compassionate healthcare in general (Malenfant et al., 2022; Sinclair et al., 2016). Our study has shown that women appreciate when healthcare professionals are both emotionally sensitive as well as being perceived as confident and competent, and as being trusted to act when needed. According to Gilbert (2017), compassion implies “wise actions” or what he refers to as “skillful acting.” This suggests that the expression of compassion should vary based on the specific requirements of a given situation. In the context of our study, “wise actions” means that compassionate healthcare professionals demonstrate sensitivity and warmth while also being ready to take control and act, when necessary, based on the ability to adapt to the needs and context of each woman.

This requires not only contextual adaptation but also cultural sensitivity (Fair et al., 2020). Cultural values and norms can influence a woman’s preferences and expectations around childbirth (Small et al., 2014). A culturally sensitive approach may enable healthcare professionals to provide care that is truly tailored to each woman’s individual needs (Shorey et al., 2021). Singh et al. (2018) note that compassionate and respectful attitudes from healthcare professionals can help overcome cultural differences and compensate for a potential lack of understanding about a patient’s culture.

We would argue that, for example, small acts of kindness are dependent on a sensitivity from the healthcare professional to be experienced as compassion. For instance, attuning actions can ensure that healthcare professionals remain sensitive to the woman’s signals and needs, enabling constant adjustments to provide compassionate care. Thus, the five themes can represent the balance between being both sensitive to the woman’s needs and vulnerability and at the same time showing a motivation to try to alleviate and prevent the woman’s challenges, needs, and suffering.

The description of actions that are perceived compassionately may contribute to reflections around healthcare professionals’ own practices, as well as teaching and developing compassionate skills for midwifery and medical students during their training. However, this requires that healthcare professionals are not constrained by their work environment, that they have time, and that there is a culture and environment acknowledging the system-level issues associated with the provision of compassionate care (Cole-King & Gilbert, 2014). According to Valizadeh et al. (2018), the ability for healthcare professionals to provide compassionate care may be affected by factors such as workload, staffing levels, and organizational culture. For instance, a high-stress work environment with overwhelming patient loads and limited time may hinder healthcare professionals to provide the level of compassionate care they desire and further lead to compassionate fatigue (Sinclair et al., 2017). In contrast, a supportive compassionate work environment may improve the capacity of healthcare professionals to provide compassionate care (Crowther et al., 2019).

### Strengths and Limitations

The study provides new insights into a more detailed and in-depth understanding of what is perceived as healthcare professionals’ compassionate caring actions. The sample was diverse in several aspects, as it included women of different parity, birth experiences, modes of birth, medical interventions, and complication, as well as homebirths and hospital births. In addition, the study is informed by a theoretical conceptualization of compassion, which enhances the interpretations of the findings. Lastly, it is of clinical relevance to healthcare providers who encounter women during their maternity episodes because it elaborates on concrete compassionate attributes, behaviors, and actions recognized by the users of maternity care services.

However, there are several limitations to this study. First, we acknowledge that including more seldom-heard groups in the sample could have provided greater transferability of the findings. These groups are particularly at risk of experiencing uncompassionate care (Bohren et al., 2015; Vedeler et al., 2023). We did not specifically ask participants about their ethnicity, culture affiliation, or gender identity, which can be seen as a limitation of the study. However, attempts were made to include women with different backgrounds and childbirth experiences, which resulted in a sample representing various experiences of maternity care in Norway. Second, although we gained a large amount of information, for some of the women, it was challenging to recall the healthcare professionals’ concrete actions. It appeared that some of the participants found it easier to describe their feelings when cared for compassionately. Other prompts or data collection methods or a different timing of data collection closer to birth may have contributed to easier recollection of such actions, if they existed.

Further research should focus on including more diverse groups such as ethnic and gender minorities and socioeconomically deprived women.

## Conclusion

The present study provides evidence on how women in Norway perceive compassionate care from healthcare professionals. It could contribute to understanding more of the meaning of compassion during childbirth for childbearing women and to the kinds of actions that express compassionate caring. Women perceive the “doing” of compassionate care by healthcare professionals as attuning, validating, contextualizing, and empowering actions and small acts of kindness. The current study shows the importance of compassionate care as wise skillful actions that are placed at the very core of maternity care services.
